# Supplementary material for: Evidence of the interplay of genetics and culture in Ethiopia
Source: Nat Commun. 2021 Jun 11;12:3581. doi: 10.1038/s41467-021-23712-w (PMC8196081; doi:10.1038/s41467-021-23712-w)
Supplement: Supplementary file 3 — Description of Additional Supplementary Files [file 41467_2021_23712_MOESM3_ESM.pdf]

Description of Additional Supplementary Files

File Name: Supplementary Data 1
Description: Features of the 1,214 Ethiopian samples included in this work, including ethnicity, language group and self-reported first and second languages (n = sample size). “Language group” is given at the first (“Nilo-Saharan”) and to the second (other languages) classification level at www.ethnologue.com; all Nilo-Saharan speakers are from the “SatelliteCore” second classification. Labels in parentheses of column 1 are used in Figure 5; labels in parenthesis of column 3 are used in Supplementary Figure 7ab.

File Name: Supplementary Data 2
Description: Sampled groups' alternative names.

File Name: Supplementary Data 3
Description: Present-day non-Ethiopian samples included in this work, for both the “Ethiopia-internal” and “Ethiopia-external” analyses. For Region, NA=North Africa, WA=West Africa, CA=Central Africa, EA=East Africa, SO=Somalia, SA=South Africa, EG=Egypt, WE=West Eurasia, SS=South Asia, ES=East Asia, CS=Central Asia/Siberia, OC=Oceania, AM=Americas.

File Name: Supplementary Data 4
Description: Inferred fineSTRUCTURE clusters. *indicates populations not used as surrogates in the GLOBETROTTER “Ethiopiainternal” analysis, because they contained people from several ethnic groups and would hence confuse interpretation of results. “Finestructure certainty” measures the average certainty (out of 100%), across 100 MCMC iterations, for which individuals in the given cluster were assigned to that cluster, with overall average given at bottom (see Methods).

File Name: Supplementary Data 5
Description: Average genetic similarity (1-TVD) among all pairwise comparisons of individuals from different labeled groups, under the “Ethiopia-internal” analysis. Parentheses give p-values testing the null hypothesis that individuals from the row group are no more genetically similar to each other on average than individuals from this group and the column group, without adjusting for multiple comparisons. The third and fourth columns from the right give the most genetically similar group overall, under the regular analysis and under an alternative approach (**) where each Ethiopian was matched to the same numbers of individuals across groups defined in the painting (see Methods). The last two columns give other groups that are not significantly more different than the most genetically similar group under each of these approaches.

File Name: Supplementary Data 6
Description: Average genetic similarity (1-TVD) among all pairwise comparisons of individuals from different labeled groups, under the “Ethiopia-external” analysis. Parentheses give p-values testing the null hypothesis that individuals from the row group are no more genetically similar to each other on average than individuals from this group and the column group, without adjusting for multiple comparisons. Last two columns give the most genetically similar group and other groups not significantly more different than this group is to the row group.

File Name: Supplementary Data 7
Description: Details of GLOBETROTTER and SOURCEFIND inference under the “Ethiopia-external” analysis, testing whether Ethiopian clusters can be described as descending from mixtures of sources represented by non-Ethiopian groups. Clusters are named/numbered in first column as in Figure 3.

event.type: indicates whether cluster is classified into 6 types of event defined based on which sources were inferred to intermix (see main text, SI section 5). conclusion: whether GLOBETROTTER inferred “one-date” involving a single purse of admixture between two sources, (2) “one-date-multiway” involving a single pulse of admixture between >2 sources, (3) “multiple-dates” involving >1 pulse of admixture at different times (potentially between >2 sources) or (4) “uncertain” where inference is challenging to categorize into (1)-(3). language: number of individuals in the cluster whose ethnicity is from one of 4 language families (AC = Afroasiatic Cushitic, AO = Afroasiatic Omotic, AS = Afroasiatic Semitic, NS = NiloSaharan) or 2 unclassified groups (UN). pop.description: individuals per group label within cluster. r2.one: fit of inferred pulse of admixture to data (0-1 scale). two.date.score: evidence of >1 pulse of admixture (0-1 scale). fit.quality1: fit of single inferred admixture date between two sources (0-1 scale). fit.quality2: fit of more complicated admixture (0-1 scale). first.date: inferred date (gen from present + CI) of most strongly signalled admixture event. firstevent.prop: admixture proportion contributed by minor admixing source in most strongly signalled admixture event. firstevent.source1: surrogate that is the best genetic representative of the minor admixing source in the most strongly signalled admixture event. firstevent.source2: surrogate that is the best genetic representative of the major admixing source in the most strongly signalled admixture event. firstevent.source1.detailed: mixture of surrogates (and mixture proportions) of groups that best describe minor admixing source in the most strongly signalled admixture event (includes all surrogates contributing >5% to mixture). firstevent.source2.detailed: mixture of surrogates (and mixture proportions) of groups that best describe major admixing source in the most strongly signalled admixture event (includes all surrogates contributing >5% to mixture). second.date: inferred date (gen from present + CI) of least strongly signalled admixture event, for clusters with conclusion = “multipledates”. secondevent.prop: admixture proportion contributed by minor admixing source in least storngly signalled admixture event, for clusters with conclusion = “one-date-multiway” or “multiple-dates”. secondevent.source1: surrogate that is the best genetic representative of the minor admixing source in the least strongly signalled admixture event, for clusters with conclusion = “one-date-multiway” or “multiple-dates”. secondevent.source2: surrogate that is the best genetic representative of the major admixing source in the least strongly signalled admixture event, for clusters with conclusion = “one-date-multiway” or “multiple-dates”. secondevent.source1.detailed: mixture of surrogates (and mixture proportions) of groups that best describe minor admixing source in the least strongly signalled admixture event (includes all surrogates contributing >5% to mixture), for clusters with conclusion = “one-date-multiway” or “multipledates”. secondevent.source2.detailed: mixture of surrogates (and mixture proportions) of groups that best describe major admixing source in the least strongly signalled admixture event (includes all surrogates contributing >5% to mixture), for clusters with conclusion = “one-date-multiway” or “multipledates”. Remaining columns give the inferred proportion of DNA for which each cluster is inferred to share most recent ancestry with non-Ethiopian individuals from given regions (Mota = 4.5kya Ethiopian).

File Name: Supplementary Data 8
Description: Details of GLOBETROTTER inference under the “Ethiopia-internal” analysis, testing for recent admixture among Ethiopian groups. Clusters are named/numbered in first column as in Figure 3, though with three additional groups added in the final rows. Legend for all columns provided in Supplementary Data 7.

File Name: Supplementary Data 9
Description: Average genetic similarity (1-TVD) among all pairwise comparisons of individuals from different language classifications, under the “Ethiopia-internal” analysis.Parentheses give p-values testing the null hypothesis that individuals from the row group are no more genetically similar to each

other on average than individuals from this group and the column group, without adjusting for multiple comparisons. The last column gives the language classifications that are not signficantly different from the row language group at a Type I error level of 0.01 (not corrected for multiple testing).

File Name: Supplementary Data 10
Description: Average genetic similarity (1-TVD) among all pairwise comparisons of individuals from different language classifications, under the “Ethiopia-external” analysis. Parentheses give p-values testing the null hypothesis that individuals from the row group are no more genetically similar to each other on average than individuals from this group and the column group, without adjusting for multiple comparisons. The last column gives the language classifications that are not signficantly different from the row language group at a Type I error level of 0.01 (not corrected for multiple testing).

File Name: Supplementary Data 11
Description File: F_ST, averaged across SNPs, among all pairs of Ethiopian groups.

File Name: Supplementary Data 12
Description File: Practices reported by different groups (blank cells = did not report)

File Name: Supplementary Data 13
Description File: For each group X={Mursi, Suri, Zilmamo} (columns), all of which traditionally wear decorative lip plates, each row gives average genetic similarity (based on TVD) between individuals from X versus those from other listed group (a) under the “Ethiopia-internal” and “Ethiopiaexternal” analyses, both using raw scores and relative to values expected under geographic distance (fit as described in Methods). I.e. values reflect averages across all pairwise comparisons of individuals, with one individual from X and the other from the group in the row. For each of X={Mursi, Suri, Zilmamo} and the average across these three, the six groups with six highest such values are shown. Letters in parentheses give the language branch classification of that ethnicity (O = Afroasiatic Omotic, N = Nilo-Saharan Satellite-Core).
