# Supplementary material for: Evidence of the interplay of genetics and culture in Ethiopia
Source: Nat Commun. 2021 Jun 11;12:3581. doi: 10.1038/s41467-021-23712-w (PMC8196081; doi:10.1038/s41467-021-23712-w)
Supplement: Supplementary file 5 — Reporting Summary [file 41467_2021_23712_MOESM5_ESM.pdf]

nature research

Corresponding author(s): Garrett Hellenthal

Last updated by author(s): Mar 31, 2021

Reporting Summary

Nature Research wishes to improve the reproducibility of the work that we publish. This form provides structure for consistency and transparency in reporting. For further information on Nature Research policies, see our Editorial Policies and the Editorial Policy Checklist.

Statistics

For all statistical analyses, confirm that the following items are present in the figure legend, table legend, main text, or Methods section.

n/a	Confirmed	
☐	☒	The exact sample size (n) for each experimental group/condition, given as a discrete number and unit of measurement
☐	☒	A statement on whether measurements were taken from distinct samples or whether the same sample was measured repeatedly
☐	☒	The statistical test(s) used AND whether they are one- or two-sided<br>*Only common tests should be described solely by name; describe more complex techniques in the Methods section.*
☐	☒	A description of all covariates tested
☐	☒	A description of any assumptions or corrections, such as tests of normality and adjustment for multiple comparisons
☐	☒	A full description of the statistical parameters including central tendency (e.g. means) or other basic estimates (e.g. regression coefficient) AND variation (e.g. standard deviation) or associated estimates of uncertainty (e.g. confidence intervals)
☐	☒	For null hypothesis testing, the test statistic (e.g. F, t, r) with confidence intervals, effect sizes, degrees of freedom and P value noted<br>*Give P values as exact values whenever suitable.*
☐	☒	For Bayesian analysis, information on the choice of priors and Markov chain Monte Carlo settings
☐	☒	For hierarchical and complex designs, identification of the appropriate level for tests and full reporting of outcomes
☐	☒	Estimates of effect sizes (e.g. Cohen's d, Pearson's r), indicating how they were calculated

Our web collection on statistics for biologists contains articles on many of the points above.

Software and code

Policy information about availability of computer code

Data collection	The Googleway v2.7.3 package in R was used to get elevation in formation.
Data analysis	conform-gt (https://faculty.washington.edu/browning/conform-gt.html)<br>Beagle 4.1 (https://faculty.washington.edu/browning/beagle/b4_1.html)<br>ATLAS (https://bitbucket.org/wegmannlab/atlas/wiki/Variant%20Discovery%20Tools:%20call)<br>PLINK v1.9 (https://www.cog-genomics.org/plink/)<br>EIGENSOFT (https://reich.hms.harvard.edu/software)<br>FastIBD in BEAGLEv3.3.2 (http://faculty.washington.edu/browning/beagle/b3.html)<br>ChromoPainterv2 (https://github.com/sahwa/ChromoPainterv2)<br>ADMIXTURE v.1.3.0 (https://dalexander.github.io/admixture/download.html)<br>fineSTRUCTURE (www.paintmychromosomes.com)<br>SHAPEIT (https://mathgen.stats.ox.ac.uk/genetics_software/shapeit/shapeit.html)<br>SOURCEFINDv2 (https://github.com/sahwa/sourcefindV2)<br>GLOBETROTTER (https://github.com/sahwa/GLOBETROTTER)

For manuscripts utilizing custom algorithms or software that are central to the research but not yet described in published literature, software must be made available to editors and reviewers. We strongly encourage code deposition in a community repository (e.g. GitHub). See the Nature Research guidelines for submitting code & software for further information.

Data

Policy information about availability of data

All manuscripts must include a data availability statement. This statement should provide the following information, where applicable:

- Accession codes, unique identifiers, or web links for publicly available datasets
- A list of figures that have associated raw data
- A description of any restrictions on data availability

Genotype data, birthplace information and self-reported group label, first language, second language and religious affiliation for newly genotyped individuals are available for non-commercial use at the European Genome-phenome Archive (EGA), which is hosted by the EBI and the CRG, under accession number EGAS00001005171.

Field-specific reporting

Please select the one below that is the best fit for your research. If you are not sure, read the appropriate sections before making your selection.

[x] Life sciences [ ] Behavioural & social sciences [ ] Ecological, evolutionary & environmental sciences

For a reference copy of the document with all sections, see nature.com/documents/nr-reporting-summary-flat.pdf

Life sciences study design

All studies must disclose on these points even when the disclosure is negative.

Sample size	Sample sizes were based on previously described analyses and simulation results (Hellenthal et al 2014, Science 343:747).
Data exclusions	Following data collection, related individuals were excluded based on using pairwise PI_HAT values inferred using PLINK v1.9. We used an outlier approach to remove related individuals within each labeled group.
Replication	No replication.
Randomization	There were no experimental groups. Ethiopian individuals were selected based on (where possible) having concordant ethnicity and birthplace information as reported for their parents and two of their grandparents.
Blinding	Blinding is not relevant -- we did use a genetics-based clustering algorithm that is blind to self-reported group label, which gave results largely consistent with group label. Given the study is looking at how group label associates with genetics, blinding is not desirable.

Reporting for specific materials, systems and methods

We require information from authors about some types of materials, experimental systems and methods used in many studies. Here, indicate whether each material, system or method listed is relevant to your study. If you are not sure if a list item applies to your research, read the appropriate section before selecting a response.

Materials & experimental systems

n/a	Involved in the study	
[x]	[ ]	Antibodies
[x]	[ ]	Eukaryotic cell lines
[x]	[ ]	Palaeontology and archaeology
[x]	[ ]	Animals and other organisms
[ ]	[x]	Human research participants
[x]	[ ]	Clinical data
[x]	[ ]	Dual use research of concern

Methods

n/a	Involved in the study	
[x]	[ ]	ChIP-seq
[x]	[ ]	Flow cytometry
[x]	[ ]	MRI-based neuroimaging

Human research participants

Policy information about studies involving human research participants

Population characteristics	We collected self-reported ethnicity, language, religious affiliation and ethnic information from each person. All individuals are anonymous. No other phenotypic information is included.
Recruitment	Participants were volunteers over the age of 18, with DNA data and (anonymized) participants' information collected via visits to Ethiopia over multiple years.
Ethics oversight	All study participants, including non-Ethiopians whose genetic variation data are newly reported in this study, gave their informed consent. Local permissions were obtained in all cases where applicable local ethical approval and regulations existed, e.g. Cameroon, Ministry of Higher Education and Scientific Research, Permits 0188/MINREST/B00/D00/D10/ D12 and 317/MINREST/B00/D00/D10 and University of Yaounde I; Ethiopia, Ethiopian Science and Technology Commission and National Ethics Review Committee. Sample collection/usage for all unpublished data included in this study were approved by the UK ethics committee London Bentham REC (formally the Joint UCL/UCLH Committees on the Ethics of Human Research: Committee A and Alpha,REC reference number 99/0196, Chief Investigator MGT). The analyses reported here were approved by UCL REC (Project ID: 5188/001).

Note that full information on the approval of the study protocol must also be provided in the manuscript.
